# scRNA-seq reveals aging-related immune cell types and regulators in vaginal wall from elderly women with pelvic organ prolapse

**DOI:** 10.3389/fimmu.2023.1084516

**Published:** 2023-02-20

**Authors:** Yali Miao, Jirui Wen, Ling Wang, Qiao Wen, Juan Cheng, Zhiwei Zhao, Jiang Wu

**Affiliations:** ^1^ Department of Obstetrics and Gynecology, Key Laboratory of Birth Defects and Related Diseases of Women and Children of Ministry of Education (MOE), West China Second University Hospital, Sichuan University, Chengdu, China; ^2^ Deep Underground Space Medical Center, West China Hospital, Sichuan University, Chengdu, China; ^3^ West China School of Basic Medical Sciences and Forensic Medicine, Sichuan University, Chengdu, China

**Keywords:** single-cell RNA sequencing, pelvic organ prolapse, aging, molecular mechanism, immune cell types

## Abstract

**Introduction:**

In the pathology of pelvic organ prolapse (POP), little is known about the contributing role of pelvic microenvironment. Also, the age-related differences in pelvic microenvironment of POP patients is always ignored. In the present study, we investigated the age-related differences in pelvic microenvironment between Young POP patients and Old POP patients, and the novel cell types and critical regulators which contributes to the age-related differences.

**Methods:**

Single-cell transcriptomic analyses were used to detect the changes in cell composition and gene expression from the pelvic microenvironment of control group (<60 years), Young POP group (<60 years) and Old POP group (>60 years). Then, immunohistochemistry and immunofluorescence were used to verify the novel cell types and critical regulators in the pelvic microenvironment. Furthermore, histopathological alteration and mechanical property alteration in POP with different ages were revealed by vaginal tissue histology and biomechanical testing.

**Results:**

The up-regulated biological process in Old women with POP is mainly related to chronic inflammation, while the up-regulated biological process in Young women with POP is mainly related to extracellular matrix metabolism. Meantime, CSF3+ endothelial cells and FOLR2+ macrophages were found to play a central role in inducing pelvic chronic inflammation. Furthermore, the collagen fiber and mechanical property of POP patients decreased with aging.

**Conclusions:**

Taken together, this work provides a valuable resource for deciphering the aging-related immune cell types and the critical regulators in pelvic microenvironment. With better understanding of normal and abnormal events in this pelvic microenvironment, we provided rationales of personalized medicine for POP patients with different ages.

## Introduction

Pelvic organ prolapse (POP), including uterine and vaginal wall prolapse caused by weakness of the supportive tissue, is a major health concern for women and can seriously impact a woman’s health and quality of life (1, 2). Among risk factors for pelvic organ prolapse, age is a key factor which is positively correlated with pelvic organ prolapse (3). Nygaard et al. investigated 1961 American women, they found that the prevalence of pelvic organ prolapse in 40~59 years old women (Young POP) was 26.5%, in 60~79 years old women (Old POP) was 36.8% (4). Another study from the United States also suggested that the number of women with POP symptoms will increase by 50% by 2050 as age increases globally (5). All the above studies suggested that the prevalence of POP increases with the aging. Although the incidence rate of POP in elderly women is high, little is known about its pathophysiological process. We need to clarify the reasons behind the fact that the elderly women are more prone to pelvic organ prolapse. Therefore, it is necessary to study the pathophysiological changes and molecular mechanism of POP based on age.

Although little is known about the factors influencing POP, patients with pelvic organ prolapse had been found to have significant alterations of the thickness, mechanical properties and structural composition of the vaginal wall (6). The alterations in histologic structure may be closely related to aging, which may partially explain the high prevalence of POP in elderly women. With aging, the collagen and elastin fibers in the pelvic microenvironment—undergoes significant changes which leads to disorders of extracellular matrix metabolism (7). Studies also shown tissue composition and biomechanical properties of the rat vagina changed in a surgically induced menopause rat model, which revealed that post-menopausal older women may be susceptible to POP development (8). On the other hand, chronic inflammation is another possible mechanism of POP. A pervasive feature of many aging tissues and age-related diseases is chronic inflammation, which is a highly significant risk factor for both morbidity and mortality in the elderly people (9). The epidemiological evidence revealed that elevated levels of inflammatory biomarkers was associated and predictive of many aging phenotypes (10). However, the etiology of inflammaging and its potential causal role in contributing to POP was little reported. The identification of specific cell type and regulators in pelvic microenvironment that control age-related inflammation across pelvic organ is therefore important in order to understand whether treatments that modulate inflammaging may be beneficial in Old POP.

Current findings about POP were mainly identified at the histological level, only partially reflecting the changes of vaginal wall in POP. The composition of vaginal wall cells in pelvic microenvironment is complex, mainly composed of fibroblasts and smooth muscle cells (11, 12). Although studies have demonstrated that aging in the vaginal wall may play a key role in the etiology of prolapse, the impact of aging on heterogeneity of vaginal cell is ignored. Hence, in order to fully understand the etiology of POP, more comprehensive and in-depth studies on specific cellular type and composition in prolapsed vaginal walls are needed to provide useful insights into the detailed molecular changes of POP.

The rapid development of single-cell RNA sequencing (scRNA-seq) technology has realized specifically analyze of cell populations and gene expression at the single-cell level, which is helpful for elucidating cell type-specific molecular alterations involved in POP. Therefore, this study will provide a comprehensive atlas of transcriptomic data for normal, young prolapsed and old prolapsed human vaginal cell types, to provide a comprehensive understanding of the molecular changes in prolapse process at the single-cell level, and verify the alterations of the mechanical properties, extracellular matrix components, and cell composition of the prolapsed vaginal wall. Finally, to offer deep understanding of the pathophysiological process of POP and new insights for diagnosis and treatment strategies of POP.

## Methods

### 10× single-cell library construction and sequencing

All tissue samples used for single-cell RNA sequencing were obtained with informed consent from all patients and the procedures in this study were reviewed and approved by the Ethics Committee of West China Second Hospital of Sichuan University. Three groups with 3 cases in each were included in this study. Postmenopausal participants with Pelvic Organ Prolapse Quantification (POP-Q) stage III in the anterior vaginal wall were divided into the following groups according to age: <60 years (Young POP), >60 years (Old POP). Exclusion criteria were chronic pelvic inflammation, chronic debilitating disease, cancer, estrogen-related diseases, connective tissue disorders and prior pelvic reconstruction surgery. Postmenopausal participant undergoing excision of vaginal cyst and without prolapse were enrolled as control group (age<60 years). Full thickness [1 cm (2)] of anterior vaginal wall tissue biopsies were harvested from the middle part of anterior vaginal wall of the control, Young POP and Old POP. The freshly separated tissue was immediately placed in α-MEM and then transported on ice to maintain viability. The vaginal wall tissue was washed with PBS for 2 to 3 times, cut into small pieces on ice, and then transferred to α-MEM digestion solution containing 1 mg/mL type I collagenase. The tissue was digested with enzyme at 37°C with a shaking speed of 150 rpm for about 80 min. The separated cells were washed with PBS and centrifuged at 600×g for 8 min to obtain cell pellets for single cell sequencing. According to the standard protocol of the 10×Genomics Chromium Single Cell 3′Kit (V3-chemistry), the single cell suspension was converted into a barcoded scRNA-seq library to capture 5000-10000 cells/chip positions. All remaining programs, including the construction of the library, are carried out in accordance with the standard manufacturer’s protocol.

The Seurat R package was used for quality control and processing of the gene expression matrix. Then, by density-based spatial clustering algorithm, cell types were identified on the tSNE map with initial setting for the eps value 0.5. In addition, differentially expressed genes (DEGs) were identified by comparing cells in a specific cluster with cells in all other clusters. And the Feature Plot function was employed to visualize the expression of a specific gene, while the DoHeat Map function was used to draw a heatmap of the marker genes in each cluster.

According to the proportion change under different conditions, the differential proportion analysis was carried out. Then, Gene ontology (GO) analysis and KEGG pathway analysis were performed to investigate the cell function status. Gene set variation analysis (GSVA) was used to evaluate the differences between subgroups of each cell type. Trajectory Analysis was used to explore the potential functional changes of different clusters. Cell-cell communication network was explored through ligand-receptor interaction.

### Immunofluorescence

Vaginal wall tissues were harvested for histology analyses according to the standard procedures. Then, immunofluorescence staining was performed to characterize CSF3^+^ endothelial cells and FOLR2^+^ macrophages. Paraffin-embedded tissue sections were deparaffinized and rehydrated in graduated alcohol, then treated in 0.1 M sodium citrate buffer and heated for 30 min for antigen retrieval. After cooling down, the endogenous peroxidase activity was blocked by 3% H_2_O_2_, and then the slides were incubated with primary antibodies respectively. Parallel controls were run with PBS. After incubation overnight, the sections were washed with PBS and then subjected to the secondary antibodies. For immunofluorescence staining, the sections were incubated by DyLight 488/549 AffiniPure-conjugated secondary antibodies (EarthOx) and counterstained with DAPI (Solarbio, s2110). The following primary antibodies were used for immunostaining: CD31 (Abcam, ab9498), CD68 (Abcam, ab213363), FOLR2 (Abcam, ab228643), CSF3 (Santa, sc-53292).

### Immunohistochemistry

To verify the inflammatory factors in pelvic microenvironment of POP patients, the expression of TNFα,IL-1β and IL-6 were investigated in 86 samples (clinical characteristics of participants were shown in **Table 1**). After pretreatment, sections were incubated with primary antibodies. Following overnight incubation, slides were washed and incubated with second antibody for 1 hour at room temperature. After another wash, the slides were counterstained with haematoxylin and visualized with the DAB. The following primary antibodies were used for immunostaining: TNFα (CST, #6945), IL-1β (CST, #27989), IL-6 (CST, #12153).

**Table 1:** Statistical analysis of clinical characteristics of participants.

Variable	Control (n=6)	Young POP (n=23)	Old POP (n=57)	*P-*value
Age, mean ± SD, years	46.5 ± 4.153	50.65 ± 1.286	71.21 ± 0.876	0.001^a^
Body mass index, mean ± SD, kg/m^2^	22.796 ± 2.092	23.918 ± 0.499	23.251 ± 0.458	0.672^a^
Hypertension, n (%)	0 (0%)	3 (13%)	23 (40.4%)	0.014^b^
Diabetes, n (%)	0 (0%)	0 (0%)	10 (17.5%)	0.056^b^
hyperthyroidism, n (%)	1 (16.7%)	1 (4.3%)	3.5 (0%)	0.345^b^

a One way ANOVA of the age, body mass index.

b Fisher’s Exact Test was used to compare differences in categorical variables. Statistical analysis was performed using the software package SPSS (Version 25.0, SPSS Inc., Chicago, Illinois, USA).

### Vaginal tissue histology

To reveal the histopathological alteration in POP patients with different ages, vaginal tissue histology was used to detect the tissue pathology. For the detection of tissue pathology, the vaginal tissue was sectioned, and the portions were fixed in 10% neutral buffered formalin, dehydrated, embedded in paraffin, sectioned at 5μm, and stained with hematoxylin-eosin (H&E).

### Biomechanical testing

In order to further distinguish the biomechanical characteristics of anterior vaginal wall prolapse in different age group, vaginal wall tissue from patients with anterior vaginal wall prolapse were included to undergo Biomechanical testing. The diagnosis of patients with POP was conducted according to the POP-Q stageing of the International Continence Society in 1995. In vaginal wall repair surgery, a median incision in the anterior (posterior) vaginal wall was made, and bilateral longitudinal tissue strips from the middle region of the prolapsed vaginal wall were removed for a tensile test. Ultimate stress strength, elastic modulus, stress-strain, stress relaxation, and creep were examined using an Instron 4302 universal material testing machine. Experimental data were collected automatically at room temperature by using a data acquisition system (JBK). In this study, stress was set to 0.77 MPa, tensile strength and stress-strain rates were 10 mm/min; creep loading speed was 10 mm/min and creep time was 600 s.

## Results

### Single-cell transcriptome atlas and main lineages in POP samples

**Figure 1:**
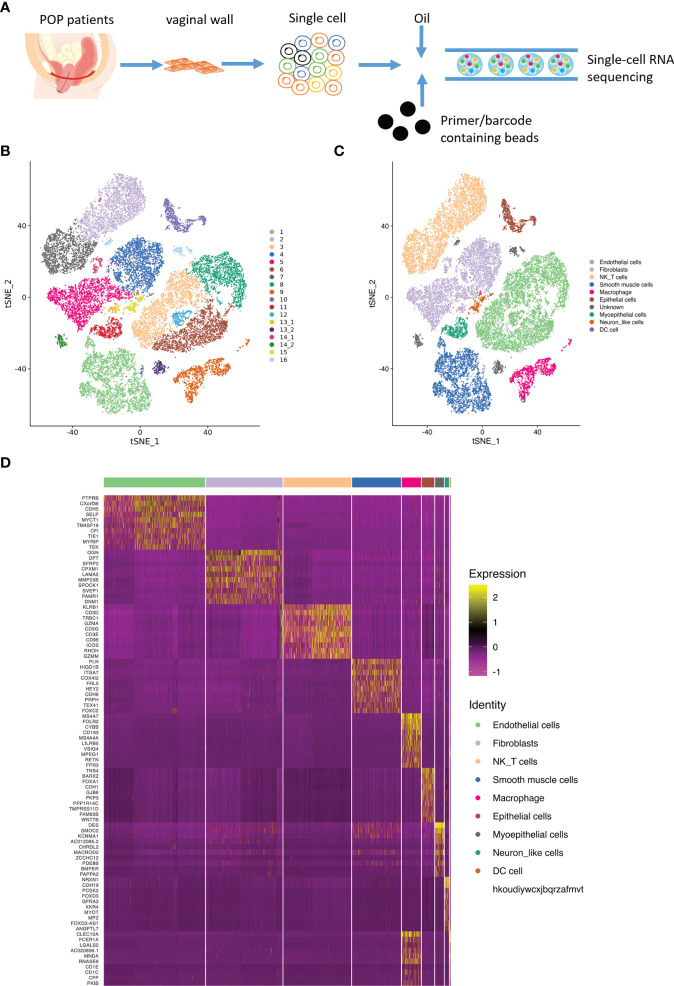
Main cell lineages in vaginal wall cells. **(A)**. Schematic of tissue dissociation, cell isolation, sequencing, and downstream bioinformatics analysis. **(B)** tSNE plots of the major vaginal wall cell populations. Each point depicts a single cell, colored according to cell population. **(C)** Definition atlas of vaginal wall cells (endothelial cells, fibroblasts, NK T cell, smooth muscle cells, macrophages, epithelial cells, myoepithelial cells, Neuron like cell, and DC cell). **(D)** Heatmap showing the relative expression of marker genes in each cell type.

Following the procedure of single cell isolation (**Figure 1A**) , we obtained a total of 33866 single cells from normal and prolapsed vaginal walls, with a median of 1774 genes per cell. Among the 33866 cells, 22952 were from prolapsed vaginal wall and 10914 were from normal vaginal wall (**Figures 1B, C**). Based on the well-known cell type markers (**Figure S1**), a total of night cell types were identified in POP and control samples: endothelial cells, fibroblasts, NK T cell, smooth muscle cells, macrophages, epithelial cells, myoepithelial cells, neuron like cell, and dendrite cells (**Figure 1D**).

### Changes of the cellular composition in POP samples

**Figure 2:**
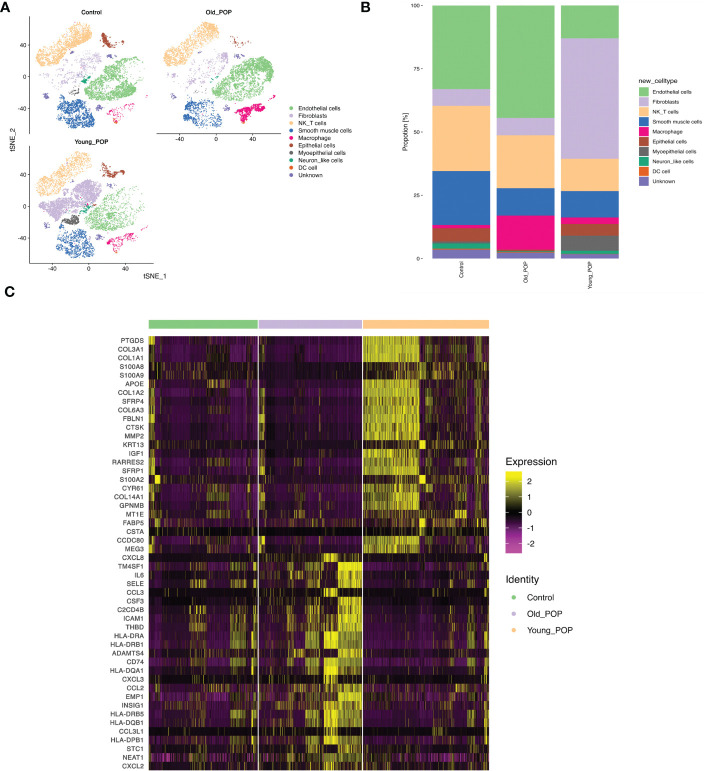
The proportions of each cluster in the Old POP, Young POP and control samples. **(A)** tSNE plots showing the distribution of cell subtypes in Old POP, Young POP and control samples. **(B)** Bar plots showing the percentage of cell subtypes in Old POP, Young POP and control samples. **(C)** The differentially expressed genes between Old POP, Young POP and control samples.

To delineate percentage changes in the cellular composition in POP, we compared scRNA-seq profiles between control, Young POP and Old POP samples. The cell types with altered proportions in the prolapsed vaginal wall are shown in **Figure 2A**. Globally, the proportion of endothelial cells and macrophage were increased in Old POP samples. Conversely, the proportion of fibroblasts and smooth muscle cells in Young POP samples were increased. Thus, the cell type-specific mechanism and age-specific mechanism in POP needs to be explored. Next, we detected differentially expressed genes between Old POP, Young POP and control sample (**Figure 2C**). Notably, Old POP sample upregulated genes related to chronic inflammation, such as IL6, CCl3, CSF3, CXCL3, CXCL2, CCL2, etc. However, extracellular matrix metabolism was activated in Young POP samples, whereas the genes COL3A1, COL1A1, COL1A2, COL6A3, MMP2, CTSK, etc upregulated. Taken together, these results indicate that dysregulation of multiple genes may be related to POP, and different age group showed different gene expression.

### Inflammation related biological processes enriched in old POP samples

**Figure 3:**
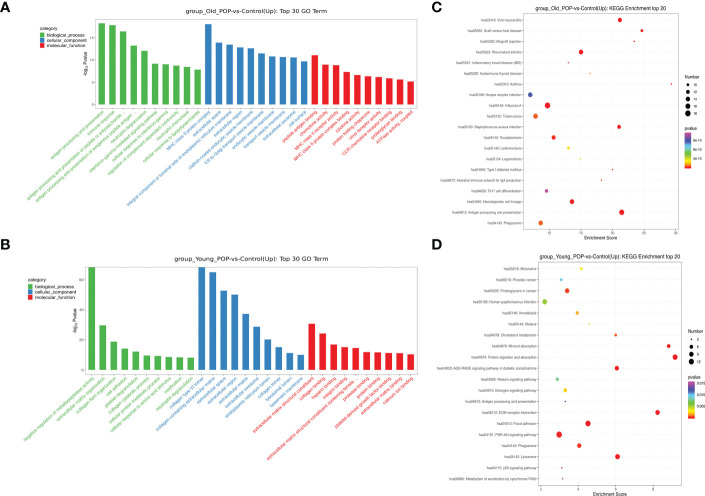
GO pathway analysis and KEGG pathway analysis in Old POP, Young POP and control samples. Histogram showing up expressed biological processes in the GO analysis of Old POP vs control samples **(A)** or Young POP vs control samples **(B)** Dot plot showing up expressed biological processes in the KEGG analysis of Old POP vs control samples **(C)** or Young POP vs control samples **(D)** X-axis, gene ratio; Y-axis, enriched biological processes terms; color (red, high; blue, low), −log10(P-value) of each term; circle size, total gene counts in each term.

Go analysis showed that the up-regulated biological process in Old POP was related to chronic inflammation such as antigen presentation, neutrophil chemotaxis and cytokine activity, while the up-regulated biological process in Young POP was mainly related to extracellular matrix metabolism such as extracellular matrix composition, collagen fiber composition and matrix metalloproteinase activity (**Figures 3A, B**). In addition, KEGG pathway analysis revealed similar results with Go analysis. As shown in **Figures 3C, D**, the up-regulated biological process of Old POP is mainly enriched in immune related diseases such as viral myocarditis, rheumatoid arthritis, inflammatory enteritis and autoimmune thyroid disease. And the up-regulated biological process in Young POP is mainly enriched in extracellular matrix related pathways such as extracellular matrix receptor and focal adhesion. Therefore, the above results suggested that chronic inflammation and extracellular matrix metabolism play an important role in the POP, and the microenviroment in POP patients of different ages is different.

### Differential expression of endothelial cells, fibroblast and macrophage in POP samples

**Figure 4:**
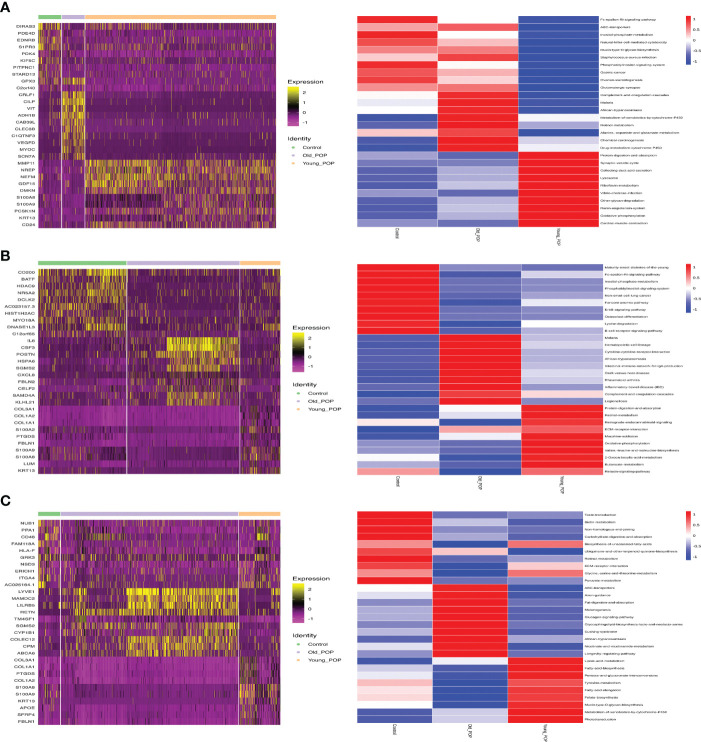
Marker genes and biological process of main cell lineages in Old POP, Young POP and control samples. **(A)** Fibroblast; **(B)** Endothelial cells; **(C)** Macrophage. The endothelial cells, fibroblast and macrophage in Old POP highly expressed inflammation related gene CSF3, CRLF1, LYVE1, separately. The endothelial cells, fibroblast and macrophage in Young POP highly expressed extracellular matrix related gene COL3A1, MMP11, S100A8, separately.

Differential gene expression of endothelial cells, fibroblast and macrophage in Old POP, Young POP and control samples were shown in **Figure 4**. The endothelial cells, fibroblast and macrophage in Old POP highly expressed CSF3, CRLF1, LYVE1 separately, which suggested an inflammation related gene enrichment. The endothelial cells, fibroblast and macrophage in Young POP highly expressed COL3A1, MMP11, S100A8 separately, which suggested an extracellular matrix related gene enrichment. In summary, we identified important candidate differential gene regulating cell functions of endothelial cells, fibroblast and macrophage in Old POP and Young POP.

### Subtypes analysis revealed CSF3^+^ endothelial cells and FOLR2^+^ macrophages were the key cell types in old POP samples

**Figure 5:**
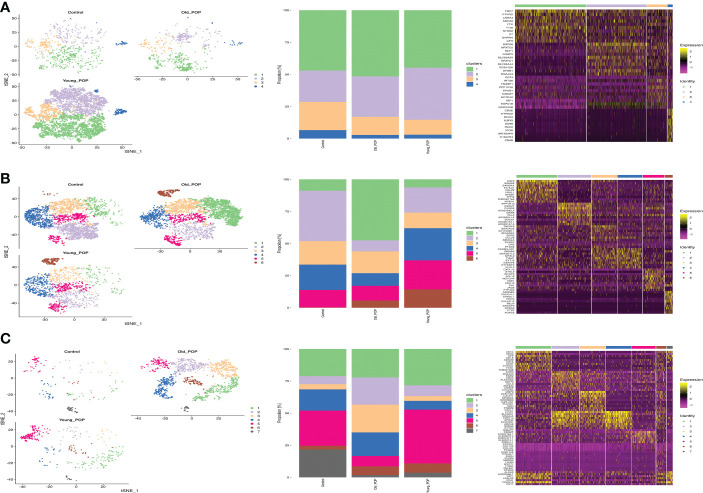
Subtypes analysis in Old POP, Young POP and control samples. **(A)** Fibroblast; **(B)** Endothelial cells; **(C)** Macrophage. tSNE map showed the distribution of distinct cell subtypes, Bar plots showed the percentage of cell subtypes in each patient, and heat map showed the relative expression of representative differential expressed gene.

**Figure 6:**
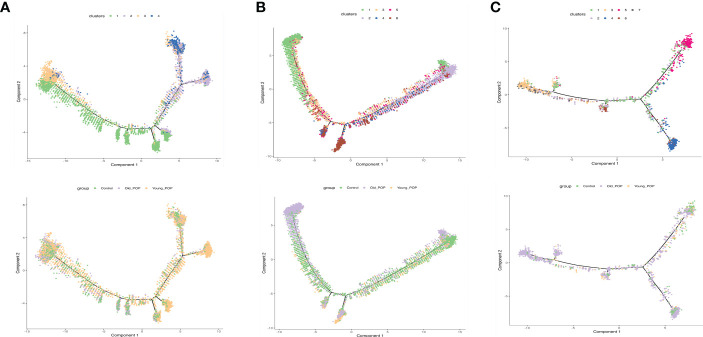
Pseudotime analysis of main cell lineages in Old POP, Young POP and control samples. **(A)** Fibroblast; **(B)** Endothelial cells; **(C)** Macrophage. In branched pseudotime trajectory, each cell was colored by its pseudotime value and its seurat clusters.

**Figure 7:**
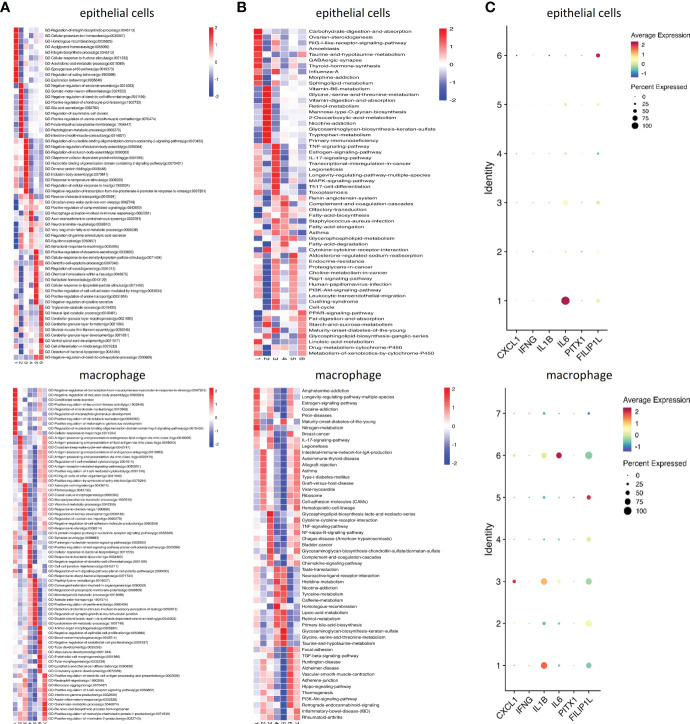
Inflammatory changes in the subtypes of endothelial cells and macrophage of the Old POP. **(A)** The heatmap showing the differences in GO terms by GSVA enrichment scores among the different subtypes. **(B)** The heatmap showing the differences in KEGG pathways by GSVA enrichment scores among the different subtypes. **(C)** The dot plots of the expression of inflammatory factors-CXCL1, IFN-γ, IL-1β, IL-6, PTX1, and FILIP1L.

We next explored the subtypes of some specific cell types. In our samples, vaginal fibroblasts were detected, and 4 distinct subtypes were acquired by subclustering (**Figure 5A**). Proportionally, subtypes 2 were strongly enriched in Young POP samples. Similarly, we classified endothelial cells into 6 transcriptionally distinct subtypes (**Figure 5B**). Proportionally, subtype 1 was strongly enriched in Old POP samples, and subtype 6 was enriched in Young POP samples. Further, macrophages could be classified into 7 transcriptionally distinct subtypes (**Figure 5C**). Proportionally, subtypes 3 were strongly enriched in Old POP samples, and subtype 5 was enriched in Young POP samples. Moreover, as shown in pseudotime analysis (**Figure 6**), the putative developmental trajectory of endothelial cells and macrophages was distinguished. the trajectory for subtype 1 of endothelial cells and subtype 3 of macrophages presents the most adjacent location to Old POP, indicating a significantly relationship to Old POP. In addition, subtype 1 of endothelial cells and subtype 3 of macrophages was closely related to inflammatory pathway and molecules (**Figure 7**), which is consistent with the previous results revealing the up regulation of inflammatory response in Old POP. Also, genes were specifically expressed in some subtypes. Subtype 1 of endothelial cells highly expressed inflammatory factor CSF3 (**Figure S2A**), which is defined as CSF3^+^ endothelial cells. Subtype 2 of macrophages highly expressed FOLR2, which is defined as FOLR2^+^ macrophages (**Figure S2B**). Therefore, these results suggest that CSF3^+^ endothelial cells and FOLR2^+^ macrophages may be closely related to POP in the elderly, and may be the cytological basis of increased inflammation in the Old POP.

### Characterizations of CSF3^+^ endothelial cells, FOLR2^+^ macrophages and inflammatory factors in POP samples

**Figure 8:**
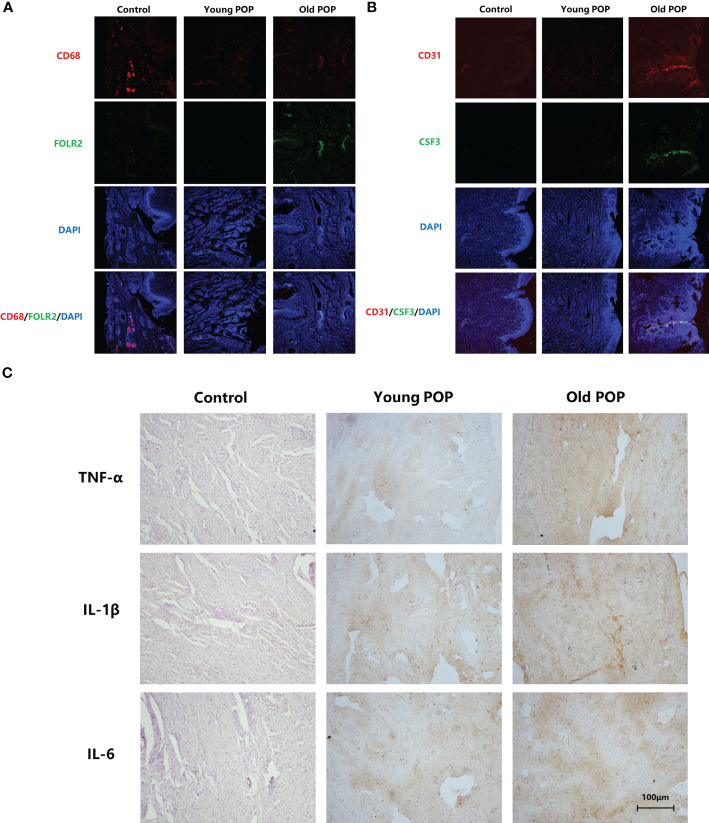
Histological characterizations of the main cell types and inflammatory changes in the Old POP. **(A)** Immunofluorescence staining for CSF3+ endothelial cells. **(B)** Immunofluorescence staining for FOLR2+ macrophages (×100). **(C)** IHC staining for TNFα, IL-1β, IL-6 (×200).

First, we labeled endothelial cells with CD31 and labeled macrophages with CD68. Then, we used CSF3 to label CSF3^+^ cells in CD31^+^ endothelial cells, and used FOLR2 to label FOLR2^+^ cells in CD68^+^ macrophages. We found double fluorescence positive CSF3^+^ endothelial cells and double fluorescence positive FOLR2^+^ macrophages significantly increased in human vaginal wall tissue of Old POP (**Figures 8A, B**). Additionally, expression of inflammatory markers TNFα, IL-1β, IL-6 in the vaginal wall tissue were verified by immunohistochemistry. Compared to control and Young POP, the expression of the TNFα, IL-1β and IL-6 was significantly upregulated in the Old POP (**Figure 8C**; **Table 2**). These data support the idea that chronic inflammation increased in the prolapsed vagina of old patients. Thus, the inflammation related mechanism in Old POP needs to be explored.

**Table 2:** Expression of TNFα, IL-1β and IL-6 in Control, Young POP, Old POP group.

	Control	Young POP	Old POP	*P-*value
TNFα score				
0	4 (66.7%)	2 (8.7%)	4 (7.0%)	0.001^a^
1	2 (33.3%)	15 (65.2%)	35 (61.4%)	0.001^a^
2	0 (0.0%)	6 (26.1%)	18 (31.6%)	0.001^a^
IL-1β score				
0	3 (50.0%)	2 (8.7%)	5 (8.8%)	0.031^a^
1	3 (50.0%)	14 (60.9%)	31 (54.4%)	0.031^a^
2	0 (0.0%)	7 (30.4%)	21 (36.8%)	0.031^a^
IL-6 score				
0	4 (66.7%)	3 (13.0%)	5 (14.0%)	0.002^a^
1	2 (33.3%)	15 (65.2%)	33 (581%)	0.002^a^
2	0 (0.0%)	5 (21.7%)	19 (27.9%)	0.002^a^

a Fisher’s Exact Test was used to compare differences in categorical variables.

### Histopathological struture and mechanical property of human vaginal wall tissues in POP samples

**Figure 9:**
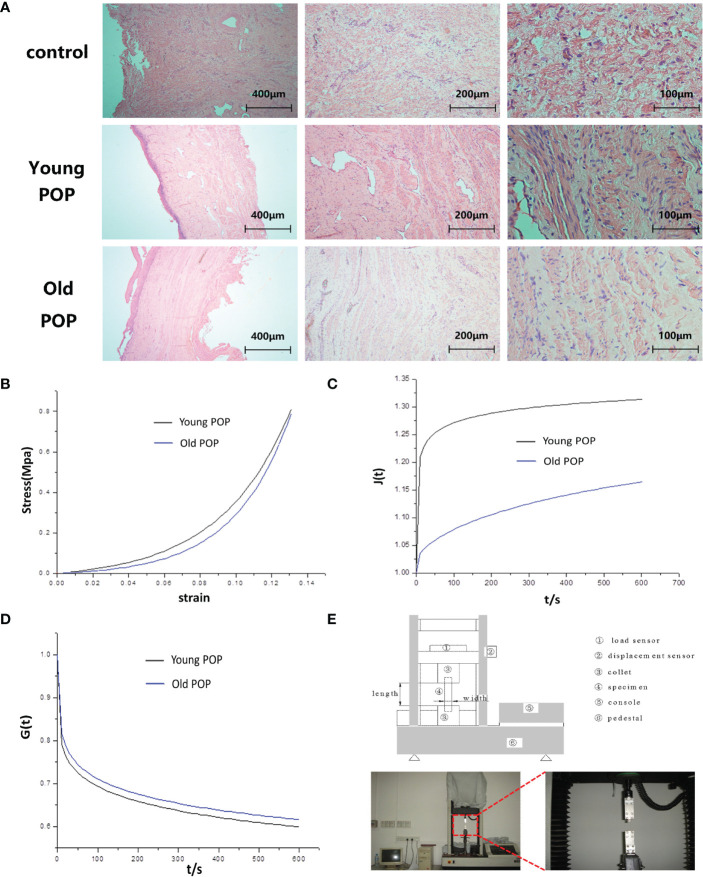
Alteration of histopathological struture and mechanical property in human vaginal wall tissues of Old POP. **(A)** HE staining showing degenerative changes in vaginal tissue of old patient with POP. In patients with pelvic organ prolapse, the collagen fibers in the vaginal tissues became sparse and lax, losing their tight junctions and showing degenerative changes. **(B)** Stress-strain diagram of the anterior vaginal wall in patients with anterior vaginal wall prolapse. **(C)** Creep performance of the anterior vaginal wall in patients with anterior vaginal wall prolapse. **(D)** Relaxation performance of the anterior vaginal wall in patients with anterior vaginal wall prolapse. **(E)** Physical picture of universal mechanical tester.

Considering that Young POP and Old POP have significantly different pathophysiological mechanisms, we speculated that the histomorphological changes in the vaginal tissues of patients with POP varied by age. From our results, we found the fibers became sparse and lax in the vaginal tissue of women with POP, and the older the patient with POP, the more sparse the collagen fibers (**Figure 9A**). In addition, we study the changes of biomechanical properties of vaginal wall tissue with increase of age. Statistical analysis showed that the elastic modulus and the ultimate stress strength of the anterior vaginal wall in the Young POP was higher than that in the Old POP (**Table 3**). The stress–strain relationship and creep performance of the anterior vaginal wall in the Young POP was higher than that in the Old POP, while the stress relaxation of the anterior vaginal wall in the Young POP was lower than that in the Old POP, which were tested by the universal mechanical tester (**Figures 9B–E**).

**Table 3:** Comparison of ultimate stress strength and elastic modulus of anterior vaginal wall in patients with anterior vaginal wall prolapse at different age groups.

Age(year)	n	Ultimate stress strength (MPa)	Elastic modulus (MPa)
50-59	7	3.8709 ± 1.78004	13.3429 ± 5.89261
60-69	6	2.4730 ± 1.60854	8.6167 ± 6.63669
70-79	13	2.4909 ± 1.68937	5.0385 ± 5.14110

## Discussion

Reduced collagen content and increased tissue expression of MMPs in samples of pelvic floor supporting tissue is considered to be an important pathological feature of POP (13–15). However, our results suggested that this pathological feature was not applicable to all POP patients. Our study confirmed the gene expression changes in Old POP, Young POP and control samples, and some specific upregulated and downregulated genes were identified. Compared to control sample, Old POP sample showed a slightly increase of collagen catabolism related genes MMP2, CTSK, a slightly decrease of collagen anabolism related genes COL3A1, COL1A1, COL1A2, but mainly upregulated genes related to inflammatory gene IL6, CCl3, CSF3, CXCL3, CXCL2, CCL2. These results implied the chronic inflammation rather than ECM organization was the main disorder of Old POP. Strikingly, extracellular matrix metabolism was activated in Young POP, whereas the expression of collagen catabolism related genes and collagen anabolism related genes both upregulated. Despite the enhancement of collagen catabolism, due to the compensatory increase of collagen anabolism, Young POP may have better tissue structure and function than Old POP.

Our study further revealed the subtle difference between Old POP and Young POP. The cells in the Old POP group were enriched with the biological pathways related to chronic inflammation (antigen presentation, neutrophil chemotaxis, cytokine activity, etc.), and the biological pathways (extracellular matrix composition, collagen fiber composition, matrix metalloproteinase activity, etc.) were enriched in the cells of the Young POP group. The results showed that the pelvic microenviroment was disordered, accompanied by increased chronic inflammation and changes of extracellular matrix components. But in Old POP group, the occurrence of pelvic organ prolapse is mainly related to the increase of chronic inflammation. While in Young POP group, the occurrence of pelvic organ prolapse is mainly related to the changes of extracellular matrix components.

Cellular and molecular changes may finally cause histomorphological changes of pelvic floor tissue. As part of the ageing process, various components of the ECM and cell matrix interactions are altered in ageing connective tissues (16). The main change in ageing connective tissues is decrease in fibrillary collagens and thin in fiber bundles (17). Our study suggested that collagen fibers in the pelvic floor tissue of patients with POP became sparse and lax, and the older the patient with POP, the more sparse the collagen fibers, which revealed the histomorphological changes of pelvic floor tissue of patients with POP. Ageing and trauma have an impact on modifying the mechanical behavior of pelvic floor tissues. Our study highlighted the importance of the biomechanical function of the vaginal wall in patients with different age of POP. The ultimate stress strength, elastic modulus, stress-strain, the creep and the stress relaxation of the vaginal wall in pelvic organ prolapse patients was significantly related to age, and these biomechanical properties decreased with age. The results contribute to the understanding of vaginal wall mechanical properties in patients with different age of POP. Results also suggested that due to the different pelvic floor tissue microenvironment, the histopathological struture and mechanical property of Old POP is indeed different fromYoung POP.

There were some limitations and strengths in our study. The limitation of our study was the small number of samples used in single cell transcriptome sequencing, which may cause deviations in actual physiological condition. Thus, precise cellular level and tissue-level experiments in the current work were developed to prove these clinically relevant studies. The strength of our study was the use of the Old POP sample, Young POP sample and non prolapse sample to detect the subtle differences in the POP patients with different ages, following the verification of a large number of clinical samples.

**Figure 10:**
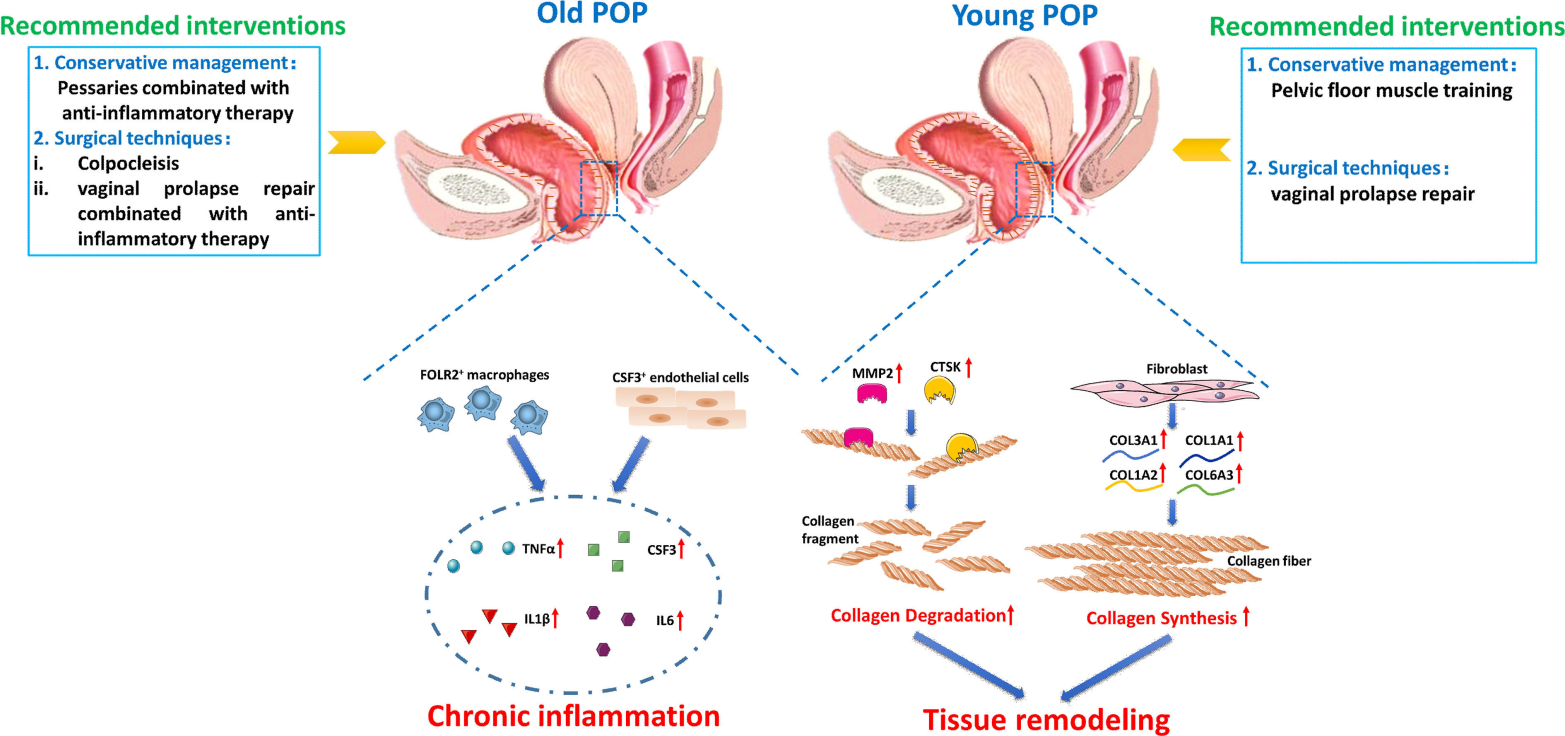
Sketch diagram unveils age-related differences between the pathology and treatment of Young POP and Old POP. The main pathology in Old POP was chronic inflammation, while the pathology in in Young POP was mainly related to tissue remodeling. These findings are helpful for improving current prevention and treatment strategies of POP, which suggest the clinicians to adopt different clinical interventions for patients with POP of different ages. For conservative management, Pessaries combinated with anti-inflammatory therapy should be given priority to Old POP, while pelvic floor muscle training can be given priority to Young POP. For surgical techniques, colpocleisis and vaginal prolapse repair combinated with anti-inflammatory therapy are preferred to Old POP, while vaginal prolapse repair could be given priority to Young POP.

Our results have important significance for the treatment of POP. Different treatment options for POP are available, including observation, nonsurgical interventions(Pessaries, pelvic floor muscle training), and surgical techniques (colpocleisis, vaginal prolapse repairs). Pessaries are a generally quite safe and well tolerated treatment option for many patients who are not candidates for surgery. However, Friedman et al. found that overall 77% women continued using a pessary for more than 1 year, and older women were more likely to continue use compared to young women (18). For women with long time vaginal pessary treatment, there is an overall complication rate of 56% including bleeding and tissue erosion, device extrusion, vaginal discharge, pain, and constipation (19). Combined with our results, we infer that the long time use of vaginal pessary and high complication rate of old women is due to the inflammatory reaction of local pelvic floor tissue. Complications of pessary use are treated with targeted therapy such as vaginal estrogen supplementation at the present study (20). Our findings highlight the need of local anti-inflammatory therapy for patients using vaginal pessaries to decrease the usage time of pessaries and help to prevent development of more serious complications. On the other hand, studies confirmed that pelvic floor muscle training showed a more prominent effect when applied as conservative management for POP (21, 22). However, the effect of pelvic floor muscle training is different in different age patients with pelvic organ prolapse. In aged person, although pelvic floor muscle training led to a significantly greater improvement in Pelvic Floor Distress Inventory-20 score, the difference between the pelvic floor muscle training group and watchful waiting groups was below the presumed level of clinical relevance (23). The non-ideal therapeutic effect of pelvic floor muscle training on Old POP may be because the occurrence of pelvic organ prolapse in the Old person is mainly related to the increase of chronic inflammation. Notably, some women must receive surgical treatment because of severe pelvic organ prolapse. Generally, old women tend to have higher complication rates and longer hospital stays than younger cohorts (24, 25). The prominent surgical complication of Old POP is urinary tract infection, which may be also related to the increase of inflammatory reaction of local pelvic floor tissue (26). So the pelvic surgeon needs to carefully choose different prolapse surgical techniques so that surgical treatment can be tailored to patient needs. Colpocleisis is an operation to prevent POP by closing the vagina, which provides a more durable outcome, minor surgical trauma and less complications than vaginal prolapse repairs in elderly women (27, 28). But for older women who still want coitus, vaginal prolapse repair can be performed with targeted therapy for inflammatory or immunological injury, to inhibit chronic inflammation and reduce surgical complications. Therefore, clinicians may need to adopt different clinical interventions for patients with POP of different ages (**Figure 10**).

Although bulk RNA-seq has revealed important biological processes linked with POP development, such as ECM remodeling, bulk RNA-seq results might not be able to determine whether these changes are intrinsic molecular changes or merely reflect changes in the ratio of cell types (29–31). In 2021, Zhu lan group used single cell sequencing to study the regulatory mechanism of POP. It is found that fibroblasts and macrophages play an important role in the disorder of extracellular matrix and immune system. The results suggest that in the pathological changes of prolapse, a variety of cells participate in the abnormal regulation of extracellular matrix and the disorder of immune reaction (32). However, this study did not indicate the age-related differences in pelvic microenvironment between Young POP patients and Old POP patients. Because the cell types may dynamically change with age in the pelvic enviroment, the effect of aging on POP needs to be further verified at the single cell level. By revealing multiple novel aging-related immune cell types and regulators, our study revealed the subtle difference between Old POP and Young POP. In our study, we revealed the Old POP was mainly related to chronic inflammation, while the Young POP was mainly related to extracellular matrix metabolism.

To our knowledge, limited study investigated the possible relationship between inflammation in the vaginal wall and POP. Chen et al. reported the presence of inflammatory cells in the levator ani muscle of women with POP, accompanied by degenerative atrophy of the muscle fibers, which was not found in women without POP (33). However, the characteristics and type of inflammatory cells have not been studied. From our results, the cell types of Old POP group, Young POP group and non prolapse group were significantly different. Among them, the number of CSF3^+^ endothelial cells and FOLR2^+^ macrophage increased significantly in Old POP group. So what are the functional characteristics of CSF3^+^ endothelial cells and FOLR2^+^ macrophages? CSF3 is a kind of inflammatory cytokines, which plays an important role in promoting the proliferation and differentiation of neutrophils, mobilizing the bone marrow cells to migrate outward and tissue, and maintaining the homeostasis of tissue microenvironment. When host infection or tissue damage occurs, CSF3 can mobilize the body to release IL-6 and IL-1β,TNFα,MMP 2 et al. SASP (34, 35). In addition, FOLR2^+^ macrophages are activated macrophages, which secrete TNF-α,IL-12, IL-6, CXCL1, iNOS, etc, causes local tissue dysfunction (36). Therefore, in Old POP, CSF3^+^ endothelial cells and FOLR2^+^ macrophages were the cytological basis of the increased chronic inflammation. Inflammatory cells can engage in complex interplay with resident non-immune cells and the ECM of tissue during tissue injury. It has been found that in the local cellular microenvironment of pelvic floor tissue, the expression of inflammatory factor in the anterior vaginal wall of POP patients increased, which affected collagen metabolism, and anti-inflammatory treatment was helpful to treat POP (37, 38). Our results provide more targeted cell types and molecules for the anti-inflammatory treatment of Old POP.

## Conclusions

In general, this study provides a comprehensive single cell transcriptome profile for deciphering gene expression profiles of heterogeneous cell types in the vaginal wall of POP, which broadens our understanding of cell characteristics and cell type specific gene changes in POP. We revealed CSF3^+^ endothelial cells, FOLR2^+^ macrophages and inflammatory factors increased in Old POP. Furthermore, we also found that the change of intercellular communication may lead to the disorder of normal cell function. Also, we found that the up-regulated biological process in Old POP was related to chronic inflammation, while the up-regulated biological process in Young POP was mainly related to extracellular matrix metabolism. Finally, we found the histopathological struture and mechanical property of POP decreased with age. Therefore, these findings revealed the subtle difference between POP patients with different ages, which suggested the clinicians to adopt different clinical interventions for patients with POP of different ages.

## Data availability statement

The datasets presented in this study can be found online in Wen, Jirui (2022): scRNA-seq reveals aging-related immune cell types and regulators in vaginal wall from elderly women with pelvic organ prolapse. figshare. Dataset. https://doi.org/10.6084/m9.figshare.21679364.v1.

## Ethics statement

The studies involving human participants were reviewed and approved by Ethics Committee of West China Second Hospital of Sichuan University. The patients/participants provided their written informed consent to participate in this study.

## Author contributions

Conceptualization: YM and JWu. Methodology: YM, JWe, ZZ and JWu. Formal analysis: JWe, LW and QW. Investigation: JWe, LW and QW and JC. Writing-original draft preparation: YM, JWe, ZZ and JWu. Writing-review and editing: ZZ and JW. All authors contributed to the article and approved the submitted version.
