# Supplementary figures and images for: scRNA-seq reveals aging-related immune cell types and regulators in vaginal wall from elderly women with pelvic organ prolapse
Source: Front Immunol. 2023 Feb 20;14:1084516. doi: 10.3389/fimmu.2023.1084516 (PMC9986331; doi:10.3389/fimmu.2023.1084516)

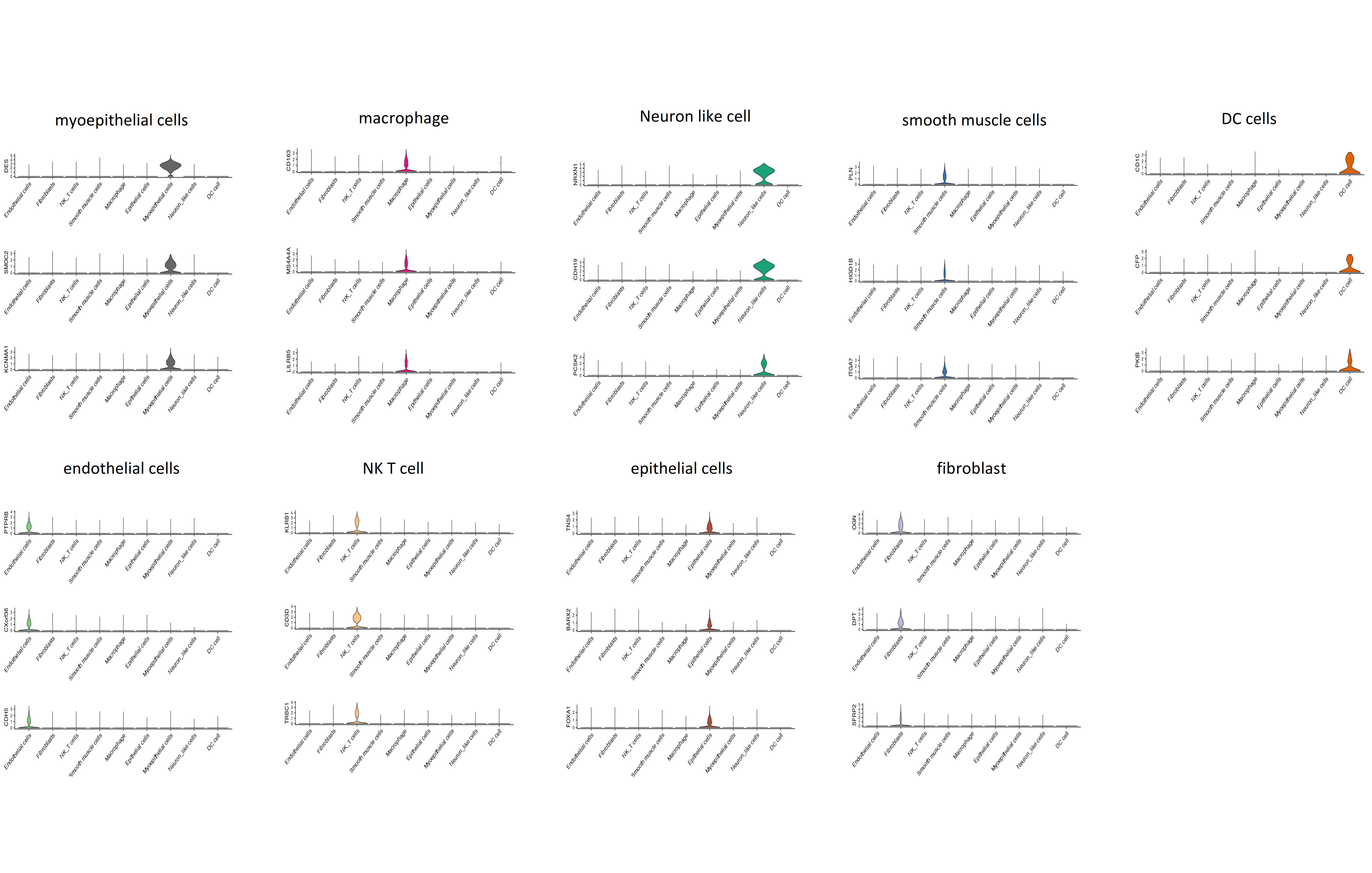

Supplement: Supplementary file 1 [file Image_1.jpeg]

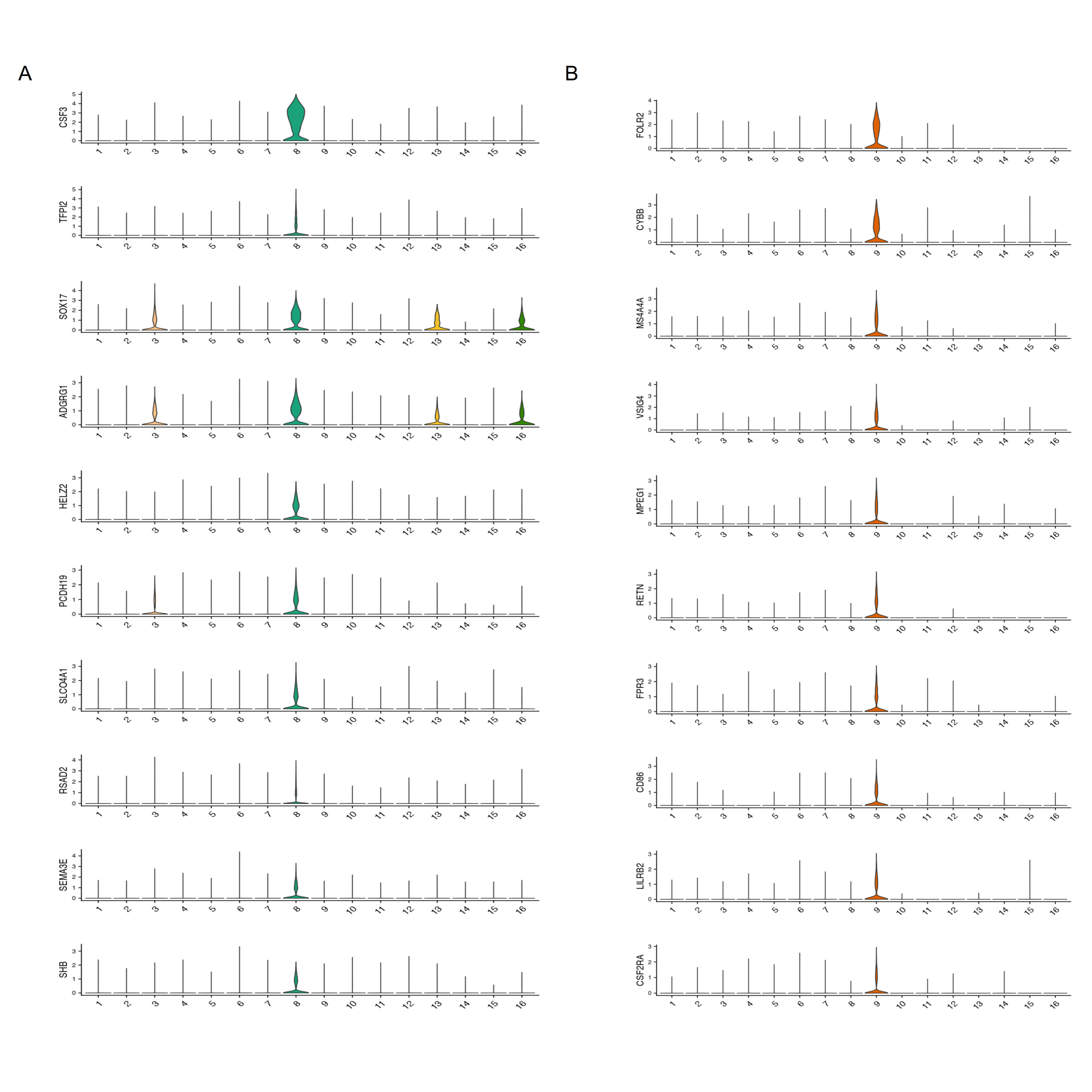

Supplement: Supplementary file 2 [file Image_2.jpeg]
